# Evidence of *Bacteroides fragilis* Protection from *Bartonella henselae*-Induced Damage

**DOI:** 10.1371/journal.pone.0049653

**Published:** 2012-11-15

**Authors:** Linda Sommese, Chiara Pagliuca, Bice Avallone, Rossana Ippolito, Amelia Casamassimi, Valerio Costa, Roberta Colicchio, Raimondo Cerciello, Maria D'Armiento, Margherita Scarpato, Alfonso Giovane, Gabiria Pastore, Teresa Infante, Alfredo Ciccodicola, Carmela Fiorito, Francesco Paolo D'Armiento, Paola Salvatore, Claudio Napoli

**Affiliations:** 1 Division of Immunohematology, Transfusion Medicine and Transplant Immunology, Regional Reference Laboratory of Transplant Immunology, Azienda Universitaria Policlinico, 1st School of Medicine, Second University of Naples, Naples, Italy; 2 Section of Microbiology, Department of Experimental Medicine, 1st School of Medicine, Second University of Naples, Naples, Italy; 3 CEINGE-Advanced Biotechnologies, Naples, Italy; 4 Department of Biological Science, University of Naples “Federico II”, Naples, Italy; 5 Department of Biomorphological and Functional Science, University of Naples “Federico II”, Naples, Italy; 6 Department of General Pathology and Excellence Research Centre on Cardiovascular Diseases, 1st School of Medicine, Second University of Naples, Naples, Italy; 7 CNR, Institute of Genetics and Biophysics “A. Buzzati-Traverso”, Naples, Italy; 8 Foundation-SDN, Institute of Diagnostic and Nuclear Development, IRCCS, Naples, Italy; 9 Department of Biochemistry and Biophysics, Second University of Naples, Naples, Italy; 10 Department of Sciences for Biology, Geology, and Environment, University of Sannio, Benevento, Italy; 11 Department of Cellular and Molecular Biology and Pathology “L. Califano”, University of Naples “Federico II”, Naples, Italy; Charité, Campus Benjamin Franklin, Germany

## Abstract

*Bartonella henselae* is able to internalize endothelial progenitor cells (EPCs), which are resistant to the infection of other common pathogens. *Bacteroides fragilis* is a gram-negative anaerobe belonging to the gut microflora. It protects from experimental colitis induced by *Helicobacter hepaticus* through the polysaccharide A (PSA). The aim of our study was to establish: 1) whether *B. fragilis* colonization could protect from *B. henselae* infection; if this event may have beneficial effects on EPCs, vascular system and tissues. Our *in vitro* results establish for the first time that *B. fragilis* can internalize EPCs and competes with *B. henselae* during coinfection. We observed a marked activation of the inflammatory response by Real-time PCR and ELISA in coinfected cells compared to *B. henselae-*infected cells (63 *vs* 23 up-regulated genes), and after EPCs infection with mutant *B. fragilis* ΔPSA (≅90% up-regulated genes) compared to *B. fragilis*. Interestingly, in a mouse model of coinfection, morphological and ultrastructural analyses by hematoxylin-eosin staining and electron microscopy on murine tissues revealed that damages induced by *B. henselae* can be prevented in the coinfection with *B. fragilis* but not with its mutant *B. fragilis* ΔPSA. Moreover, immunohistochemistry analysis with anti-Bartonella showed that the number of positive cells per field decreased of at least 50% in the liver (20±4 *vs* 50±8), aorta (5±1 *vs* 10±2) and spleen (25±3 *vs* 40±6) sections of mice coinfected compared to mice infected only with *B. henselae*. This decrease was less evident in the coinfection with ΔPSA strain (35±6 in the liver, 5±1 in the aorta and 30±5 in the spleen). Finally, *B. fragilis* colonization was also able to restore the EPC decrease observed in mice infected with *B. henselae* (0.65 *vs* 0.06 media). Thus, our data establish that *B. fragilis* colonization is able to prevent *B. henselae* damages through PSA.

## Introduction

Intracellular pathogens, including many different bacteria, live and replicate either within endosomal compartments or in the cytosol of diverse host cells such as macrophages, dendritic cells, neutrophils, fibroblasts, epithelial or endothelial cells or erythrocytes [1], [2].

*Bartonella henselae* belongs to a group of gram-negative facultative intracellular bacteria [3]. It is the causative agent of cat scratch disease, a human infection usually characterized by persistent regional lymphadenopathy [4], [5]. The bacterial pathogenicity and clinical manifestations depend on the immune status of the infected host [2], [6]–[8]. Noteworthy, *B. henselae* is able to adhere and invade EPCs [9] HSCs (hematopoietic stem cells) [10] other than human erythrocytes [11], [12]. These circulating progenitors had been previously shown to be resistant to the infection of other intracellular pathogens [13]. Moreover, these cells have been suggested to transport *B. henselae* to peripheral tissues, particularly to the endothelium of microcirculation where vasoproliferative disorders occur [2], [9].

On the other hand, *Bacteroides fragilis* is a gram-negative anaerobe bacterium and an integral component of the gut microflora of most mammals [14]–[18]. This intestinal commensal is capable of mediating powerful effects on the host immune system, which are mostly mediated by the capsular polysaccharide A (PSA), an immunomodulatory molecule that mediates T cell-dependent immune responses [17]–[19]. Indeed, it was shown to protect animals from experimental colitis induced by *Helicobacter hepaticus* [20] and this beneficial activity was demonstrated to require the PSA [20]. This microbial molecule can exert its effects through the immune system within both the intestinal and the systemic compartments [21]–[24].

Thus, in order to gain further insights in our understanding of how commensal organisms, such as *B. fragilis,* shape host immunity in other models, we investigated whether *B. fragilis* colonization could protect animal from *B. henselae* infection and exert beneficial effects on EPCs and vascular system.

## Results

### *B. fragilis* Internalizes Progenitor Cells in vitro and Competes with *B. henselae* during Coinfection

**Figure 1:**
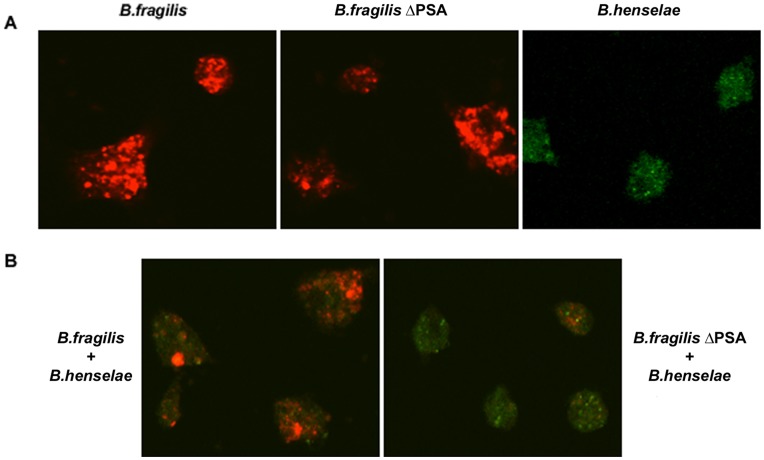
*B. fragilis* and *B. henselae* internalize EPCs. A. Confocal images of human early EPCs infected with *B. henselae, B. fragilis* and *B. fragilis* ΔPSA at 100 MOI. Cells were stained with anti-*Bacteroides* (red) or with anti-*Bartonella* (green) specific antibodies after 24 h from infection. **B.** EPCs coinfected with *B. henselae,* and *B. fragilis* or *B. fragilis* ΔPSA as indicated. A MOI of 100 was used for all bacteria strains. Cells were stained with anti-*Bacteroides* (red) and with anti-*Bartonella* (green) specific antibodies after 24 h from infection.

To evaluate the possible capacity of either *B. fragilis* or the mutant strain defective in the ability to produce PSA (*B. fragilis* ΔPSA) to adhere and invade progenitor cells, we infected *in vitro* cultured EPCs with both these bacterial strains at a multiplicity of infection (MOI) of 100. As control of infection we used *B. henselae* at 100 MOI, which has been demonstrated to elicit low cytostatic effects [9], [25]. The presence of bacteria was revealed after 24 h from infection by confocal microscopy, as shown in Figure 1A. Immmunofluorescence staining with specific antibodies showed that both *B. fragilis* and its mutant ΔPSA could internalize EPCs as previously documented for another intracellular pathogen, *B. henselae* [9]. Longer times of infection (48 h and 72 h) as well as higher MOIs showed similar results (see Figure S1–S2). Thus, we have used a MOI of 100 and 24 h of infection for further experiments.

We also carried out coinfection experiments with *B. fragilis,* or *B. fragilis* ΔPSA, and *B. henselae* on *in vitro* cultured EPCs (Figure 1B). A MOI of 100 was used for all bacteria strains. Interestingly, in the coinfection with *B. fragilis* a prevalence of Bacteroides was observed whereas in the coinfection with *B. fragilis* ΔPSA the presence of Bartonella was more evident (Figure 1B). These findings suggest *B. fragilis,* but not *B. fragilis* ΔPSA, prevents - or at some extent impairs – the internalization of *B. henselae*. Cells were observed after 24 h, 48 h and 72 h (Figure S2).

Overall, these findings clearly demonstrate that both *B. fragilis* and *B. fragilis* ΔPSA are able to internalize EPCs independently of PSA. However, only *B. fragilis* but not *B. fragilis* ΔPSA is able to partially prevent Bartonella internalization.

### Analysis of Inflammatory Chemokines and Cytokines

**Figure 2:**
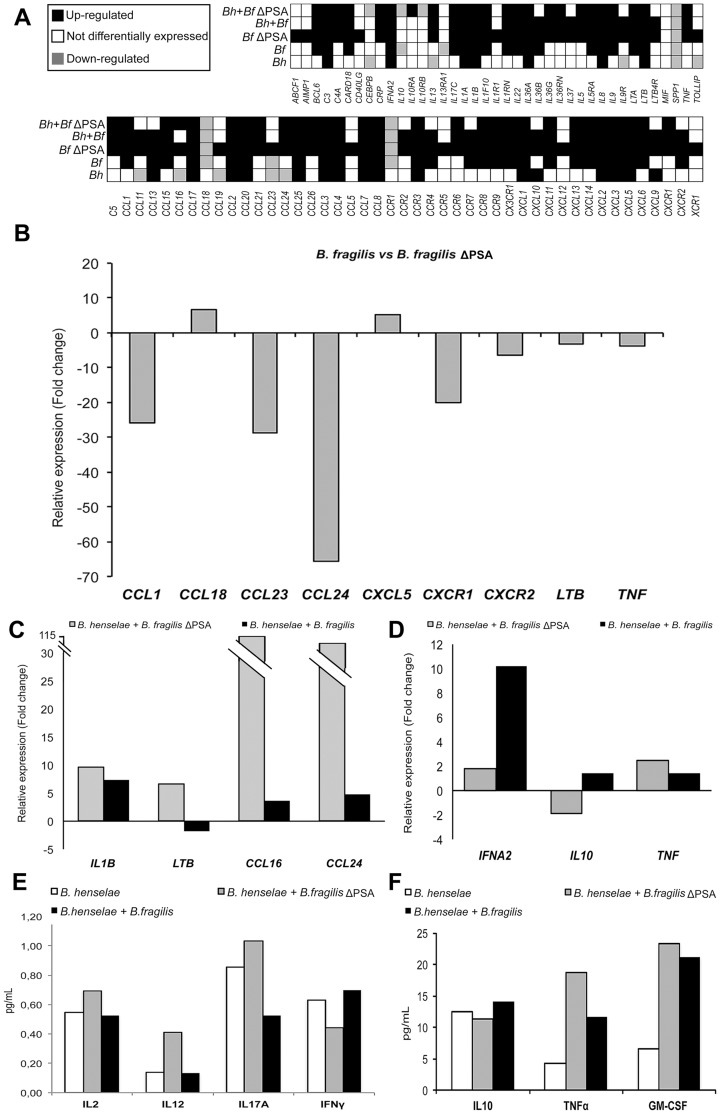
Expression levels of inflammatory genes in infected EPCs. A. Expression levels of 84 inflammatory genes in EPCs infected with *B. henselae, B. fragilis, B. fragilis* ΔPSA, *B. henselae* and *B. fragilis, B. henselae* and *B. fragilis* ΔPSA. Differential expression data are evaluated *vs* uninfected EPCs. **B.** Bar graph showing the expression of some relevant differentially expressed genes encoding chemokines, cytokines and their receptors in *B. fragilis- vs B. fragilis* ΔPSA-infected EPCs. Data are shown as relative expression levels (*B. fragilis* ΔPSA-infected cells = 1). **C.** and **D.** Expression levels of representative pro-inflammatory (C) and anti-inflammatory (D) genes highly differentially expressed during coinfection of EPCs, in presence and in absence of PSA. **E.** and **F.** Absolute quantification of relevant secreted inflammatory proteins detected by ELISA in the EPCs infected with *B. henselae,* and coinfected with *B. fragilis* and *B. fragilis* ΔPSA.

To analyze gene expression changes occurring in cultured EPCs infected by *B. fragilis* and *B. henselae*, and to assess the effect of ΔPSA on the transcription of inflammation-related genes, we used RT^2^ Profiler PCR Arrays to simultaneously profile 84 inflammatory genes encoding chemokines, cytokines and their receptors, as recently performed by Sumegi J. et al. [26]. The degree of differential expression for all the inflammatory genes within all analyzed samples is schematized in Figure 2A.

Surprisingly, even though in *B. henselae*-infected EPCs about 30 (out of 84) inflammatory genes were differentially expressed (DE) compared to uninfected cells, the highest percent of DE genes was detected in *B. fragilis* ΔPSA-infected cells (Figure S3). Particularly, in these cells 78 inflammatory chemokine and cytokine encoding genes (about 93% of all analyzed genes) were DE (Figure S3). Of note, 75 out of 78 DE genes were significantly up-regulated. On the opposite, infection with the wild-type strain of *B. fragilis* (i.e. with intact PSA) led to a decreased number of up-regulated inflammatory genes (43 *vs* 75) and a marked reduction in the expression levels of some crucial genes (Figure 2B).

In addition, we also evaluated the expression of inflammatory genes during coinfection with *B. henselae*-*B. fragilis* and *B. henselae*-*B. fragilis* ΔPSA compared to the infection with *B. henselae* alone. We observed an increased number of DE genes during both coinfections even though the extent of differential expression of inflammatory genes was more pronounced in *B. henselae*-*B. fragilis* ΔPSA cells. In particular, a direct comparison between the expression levels in the coinfected samples revealed that *B. fragilis* exerts a significant down-regulation of pro-inflammatory chemokines (shown in Figure 2C) simultaneously providing a positive modulation of anti-inflammatory genes such as *IL-10* and Interferon-alpha 2 (*IFNA2*) (Figure 2D).

We also analyzed an array of secreted cytokines involved in the inflammation response by enzyme-linked immunosorbent assay (ELISA) (Figure 2E–2F and Figure S4) on the supernatants of the same infected cells. For most of analyzed cytokines we observed a similar trend of differential expression as observed in the analysis of gene expression levels, even though the extent of DE was different. In particular, similarly to the results of RT^2^ Profiler PCR Arrays analysis, in coinfected samples the wild-type strain of *B. fragilis* exerts a significant down-modulation of pro-inflammatory interleukins (Figure 2E) and, on the opposite, a positive increase of *IL-10*, shown to repress *in vivo* the expression of TNFα and Granulocyte macrophage colony-stimulating factor (GM-CSF) (Figure 2F).

Overall, these data indicate a marked activation of the inflammatory response in *B. fragilis* ΔPSA-infected cells, reflecting both the high ability of such intracellular bacteria to invade EPCs and possibly the more rapid cell division compared to *B. henselae.* Moreover, such data confirm the role of PSA in modulating the inflammatory response, by both repressing and inducing several chemokine/cytokine-encoding genes, during *in vitro* infection of cultured EPCs.

### Murine Infection and Detection of Bacteria in Tissues from Infected Mice

**Figure 3:**
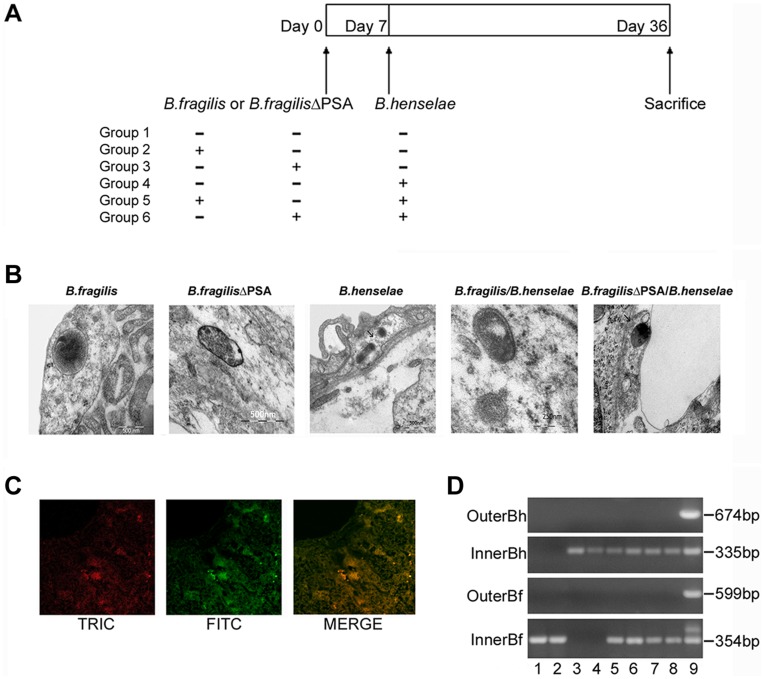
Murine infection with *B. henselae* and *B. fragilis* and detection of bacteria in infected tissues. A. Scheme of murine infection: three groups of C57BL/6J mice (n = 5) were infected with *B. henselae*, *B. fragilis*, *B. fragilis* ΔPSA respectively and two groups of animals (n = 5) were coinfected with *B. henselae* and *B. fragilis* or *B. fragilis* ΔPSA. **B.** TEM micrographs of the liver of C57BL/6J mice infected with *B. fragilis* and *B. fragilis* ΔPSA respectively, *B. henselae* and coinfected. **C.** Immunofluorescence analysis of the livers of animals infected with *B. henselae*, *B. fragilis* and coinfected. Livers were sectioned and treated with an anti-*B. henselae* and a *Bacteroides* LPS primary antibody followed by TRITC- or FITC-conjugated secondary antibodies respectively. Liver sections from uninfected mice were used as controls (data not shown). Selected merge images of coinfected mice were acquired by confocal microscopy (Magnification: all, X 270). **D.** Detection of *B. henselae gltA* gene and *B. fragilis frdA* gene by nested-PCR from the liver samples of experimentally infected C57BL/6J with *B. fragilis* and *B. fragilis* ΔPSA (lane 1–2), *B. henselae* (lane 3–4), coinfected (lane 5–8), respectively. Genomic DNA extracted from *B. henselae* and *B. fragilis* was used as positive control (lane 9).

We have developed a mouse model of coinfection to investigate whether *B. fragilis* colonization could protect animal from *B. henselae* infection. In order to determine whether PSA is essential for protection against pathogenic effects of *B. henselae*, mice were inoculated with either *B. fragilis* or its mutant *B. fragilis* ΔPSA [20]. Three groups of five mice each (n = 5) were infected with 10^9^ CFU of either *B. henselae* or *B. fragilis* or *B. fragilis* ΔPSA respectively, whereas two groups of animals were coinfected with *B. henselae* and alternatively *B. fragilis* or *B. fragilis* ΔPSA (see Figure 3A). No animals died after infection and the health was evaluated once a day for the full duration of the experiment. After 36 days, animals were euthanized in order to assess both the bacterial infection and *B. fragilis* protection by histopathology and transmission electron microscopy (TEM) of infected tissues (see paragraphs below).

The presence of the above-mentioned bacteria was documented by TEM and immunofluorescence analysis, which was also used to evaluate the colocalization (fluorescein-rhodamine) of both bacteria in coinfected mice. Bacterial internalization was observed in the endothelial and subendothelial layers around centrilobular veins and veins of the portal triads. There is no evidence of the presence of bacteria within the hepatocytes or between the hepatic laminae (Figure 3B–3C).

Paraffin-embedded liver samples obtained from infected animals were also examined for the detection of DNA of both bacterial species by PCR. Nested-PCR for the *gltA* gene (encoding for citrate synthase) (353 bp) and *frdA* gene (encoding for fumarate reductase) (354 bp) revealed the presence of *B. henselae* and *B. fragilis* DNA respectively for at least 36 days in the tissues of all mice examined (Figure 3D). No bacterial DNA was isolated from control mice (data not shown).

### *B. fragilis* Protects from the Pathogenic Effects of *B. henselae* Infection in Mice

#### Morphological analysis by hematoxylin-eosin

**Figure 4:**
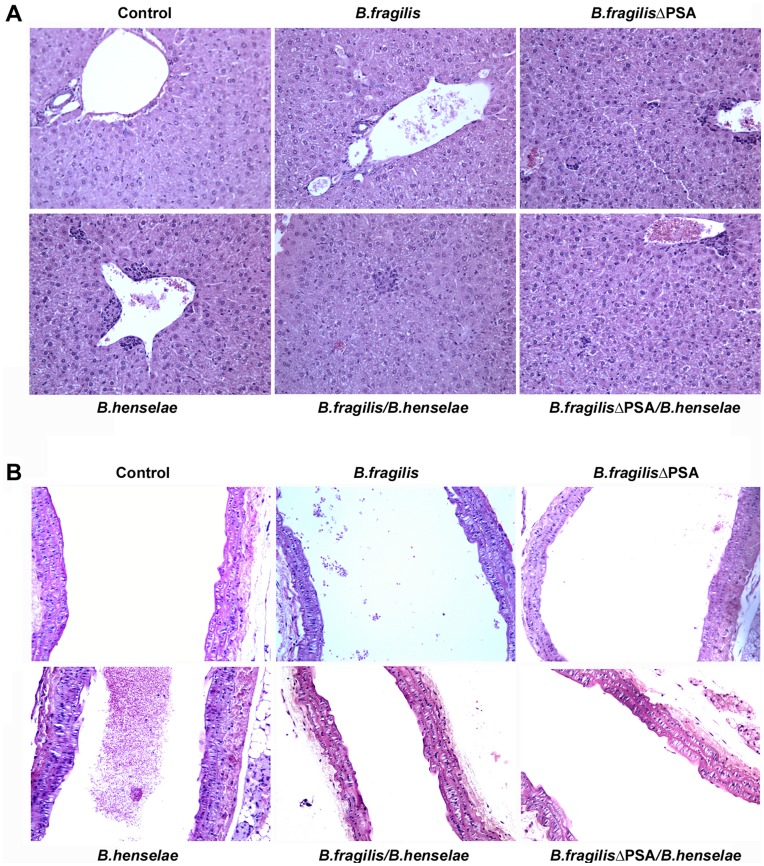
Morphological analysis of murine coinfected tissue by hematoxylin-eosin staining. A. Representative microscope images of hematoxylin-eosin staining of liver tissues from each group of mice uninfected, infected with *B. henselae*, *B. fragilis* and *B. fragilis* ΔPSA or coinfected, as detailed. Granulomatous inflammatory infiltrates are predominantly evident in the group of mice infected with *B. henselae* compared to the group infected with *B. henselae* and *B. fragilis* ΔPSA and particularly in the mice coinfected with *B. henselae* and *B. fragilis*. Uninfected controls are negative, as well as *B. fragilis* and *B. fragilis* ΔPSA groups. **B.** Representative sections stained with hematoxylin-eosin of aortas from all mice groups as described. In the group of mice infected with *B. henselae*, minimal lesions are observed in the aorta compared to liver tissues.

The morphological study performed by hematoxylin-eosin staining showed the presence of several alterations in both aorta and liver of mice infected with *B. henselae* compared to the control uninfected tissues. In the liver, we observed hydropic degeneration, nuclear pycnosis, hepatocytes with granular cytoplasm, activation of Kupffer cells and a granulomatous inflammatory reaction, as described in the literature (Figure 4) [27], [28]. These alterations were localized predominantly in the perivascular region, in the form of histioid aggregates with a mild lymphocytic infiltration (Figure 4A). Interestingly, the granulomatous infiltrates were less invasive in the group coinfected with *B. henselae* and *B. fragilis* ΔPSA and almost absent in the group of mice coinfected with *B. henselae* and *B. fragilis*. This was confirmed by the cytopathic effect and the inflammatory reaction (Figure 4A). Uninfected control, *B. fragilis* and *B. fragilis* ΔPSA groups were negative for all the described aspects. In the aorta, we documented minimal lesions in *B. henselae* infected mice. Particularly, focal endothelial swelling, with aspects of hydropic degeneration and hyalinization of basal membranes were observed (Figure 4B). These changes tend to disappear in the group of mice infected with *B. henselae* and *B. fragilis* ΔPSA and - more evident - in the aortas of *B. henselae* and *B. fragilis* infected mice (Figure 4B). In the spleen of *B. henselae* infected mice we observed an expansion of the white and red pulp, with a consequent activation of the reticulo-endothelial system (see Figure S5). In mice coinfected with *B. henselae* and *B. fragilis* ΔPSA a reduced inflammatory reaction was observed, with a decreased reactivity of the reticulo-endothelial system. A dramatic reduction in the inflammatory reaction was even more evident in *B. henselae* and *B. fragilis* infected mice.

#### Immunohistochemistry analysis

**Figure 5:**
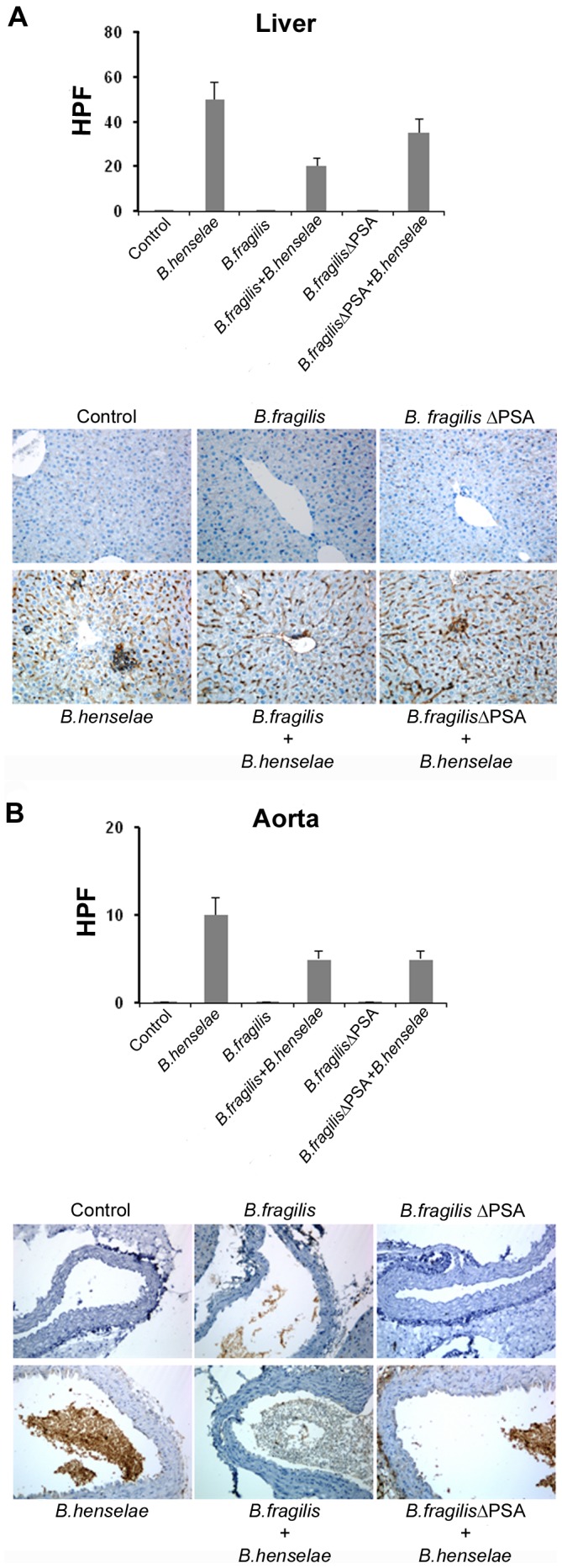
Morphological analysis of murine coinfected tissue by immunohistochemistry. A. Representative images of immunohistochemistry analysis of murine liver samples with an antibody against *B. henselae* are shown (206X magnification). Bar graphs show the mean of the positive cell number per field (HPF) as indicated. 20 fields per each section were analyzed and bars represent standard deviations. **B.** Immunohistochemistry analysis of murine aorta samples.

To confirm the morphological observations we performed immunohistochemistry assays on the liver, aorta and spleen with an anti-Bartonella specific antibody. We determined the relative count of positive cells per field (HPF) in all analyzed sections (20 fields per each section). Figure 5 schematically shows the results of such HPF counting for mice infected with *B. henselae*, *B. henselae*/*B. fragilis* and *B. henselae*/*B. fragilis* ΔPSA.

In the liver parenchyma of *B. henselae* infected mice, positive immunoreactivity to the specific antibody (i.e. infected cells) was equal to 50±8 positive cells × HPF (Figure 5A). This positivity decreased in the group of mice infected with *B. henselae* and *B. fragilis* ΔPSA (35±6 × HPF) (Figure 5A), and further reduced of about 40% in the mice coinfected with *B. henselae* and *B. fragilis* (20±4 × HPF) (Figure 5A). Few granulomatous lesions were observed, mainly concentrated in the parenchyma near the limiting pericentral laminae and, in the area adjacent to the vessels. Focal positivity in perisinusoidal areas was present. Infected cells were also detected in the granulomatous formations.

In the aorta, the percentage of *B. henselae* infected endothelial cells was minimal, with a count of positive cells of 10±2 × HPF (Figure 5B). The endothelium appeared swollen with a slight fibrous sub-endothelial layer. *B. henselae* positivity was very laborious to determine in the aortas of *B. henselae* and *B. fragilis* ΔPSA coinfected mice, with only 5±1 × HPF positive cells (Figure 5B). The same number of infected cells was also detected in the group of mice infected with *B. henselae* and *B. fragilis* (Figure 5B).

The spleen of mice infected with *B. henselae* had a cellular positivity to anti-Bartonella antibody of 40±6 cells × HPF, mainly in the red pulp. Focal positivity in the follicular centers was also evident (Figure S5). The trend of positivity reduction in both groups of coinfected mice was similar to that observed for liver and aorta tissues (Figure S5).

The decreased damage and the presence of inflammatory response in coinfected mice, particularly with *B. henselae* and *B. fragilis*, suggested us a possible pathogenic mechanism related to bacteria competition within infected cells. This assertion is corroborated by the presence of both bacteria in the cells and by a smaller cytopathic effect that attains a less inflammatory component. An intermediate state of cell damage between the groups infected with only *B. henselae* strain and those coinfected is represented by the group infected with the mutant *B. fragilis* ΔPSA. In this case a reduction of the damage is achieved compared to the *B. henselale* infected group, although to a lesser extent compared to the group of coinfected mice.

#### Ultrastructural analysis

**Figure 6:**
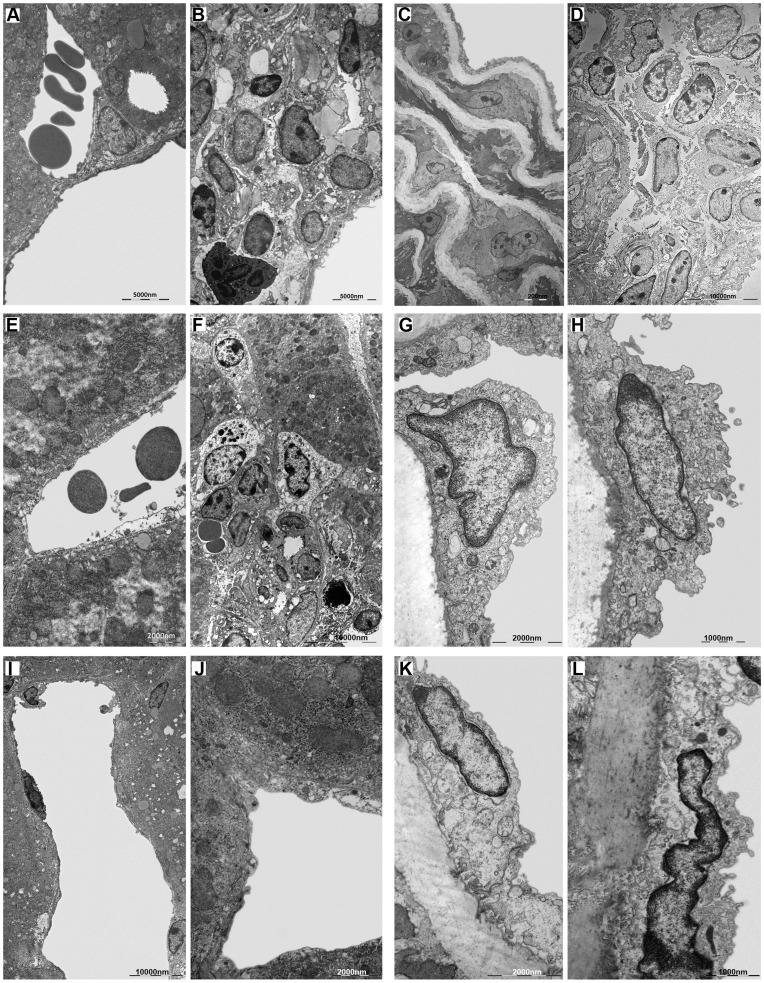
Ultrastructural analysis of murine infected tissue. Electron microscopy of liver and aorta: **A.** Portal triad of normal liver. **B.** Granuloma around a centrilobular vein of the liver infected with *B. henselae* with a characteristic neutrophil. **C.** Normal aortic intima and media. **D.** White cells in the tunica adventitia of aorta infected with *B. henselae*. **E.** Centrilobular vein of liver coinfected with *B. henselae* and *B. fragilis*. **F.** Granuloma in liver coinfected with *B. henselae* and *B. fragilis* ΔPSA. **G.** Endothelium bulging of aorta coinfected with *B. henselae* and *B. fragilis*. **H.** Endothelium bulging of aorta coinfected with *B. henselae* and *B. fragilis* ΔPSA. **I.** Centrilobular vein of liver infected with *B. fragilis*. **J.** Centrilobular vein of liver infected with *B. fragilis* ΔPSA. **K.** Endothelium of aorta infected with *B. fragilis*. **L.** Endothelium of aorta infected with *B. fragilis* ΔPSA.

The ultrastructural analysis (TEM) performed on the liver of *B. fragilis*-infected mice (Figure 6I) revealed a regular architecture of the hepatic tissue with no evidence of any inflammatory and immune response, as in the uninfected mice (Figure 6A). In contrast, the observation by TEM of livers from *B. henselae*-infected mice revealed the presence of massive granulomas, due to a *B. henselae*-induced chronic inflammatory state (Figure 6B). Such granulomas are characterized by the presence of neutrophils and few bacteria (Figure 6B and S6C), indicating a still ongoing immune response.

Surprisingly, there was any pronounced inflammatory response in the liver samples of mice coinfected with *B. henselae* and the wild type strain of *B. fragilis* (Figure 6E). Within these samples, the ultrastructural analysis revealed only few macrophages in the portal triads and around the centrilobular veins (Figure S6D_II_).

Moreover, since the role of *B. fragilis* PSA on inflammatory and immune-regulatory responses, ultrastructural analysis was also performed on the liver samples from mice infected with *B. fragilis* ΔPSA (see Methods) (Figure 6J). Such analysis revealed the presence of regular hepatic tissue architecture with no evidence of inflammatory/immune response, with the presence of active Kupffer cells in the hepatic sinusoids (Figure S6B_I_). On the opposite, in the livers of mice coinfected with *B. henselae* and *B. fragilis* ΔPSA, TEM showed again the presence of granulomas (Figure 6F), even though smaller than those observed in *B. henselae*-infected mice, confirming the role of PSA in the immunomodulation.

In all analyzed mice we did not observe the massive presence of bacteria, neither of invasomes, but few individual bacteria were visible in the space of Disse or in the granulomas (Figure S6A–E_I_).

The same ultrastructure investigations were then conducted on aorta’s sections isolated from control (Figure 6C) and infected mice (Figure 6 panels D, G, H, K and L). We did not observe the presence of granulomatous formations in any of the samples analyzed. The only exception is the presence of white cells, not organized to form a granuloma, observed in the adventitia of the aorta of *B. henselae*-infected mice (Figure 6D). Aorta, in all analyzed samples, has not significant morphological alterations, except for a marked swelling of the endothelial layer visible in *B. henselae*-infected samples (Figure S6H_II_), and in samples coinfected with *B. henselae* and *B. fragilis*, respectively wild type and ΔPSA strains (Figure 6G–6H). These bulges may be regarded as a reaction of the organ to bacteria passage, in particular of *B. henselae*, indeed, they have not been found in the aortas of mice infected with either *B. fragilis* and *B. fragilis* ΔPSA (Figure 6K–6L). Even in the aorta, we did not observe a massive number of bacteria and of invasomes, but they were found only sporadically (Figure S6F–J_I_).

#### EPC number in infected mice

**Figure 7:**
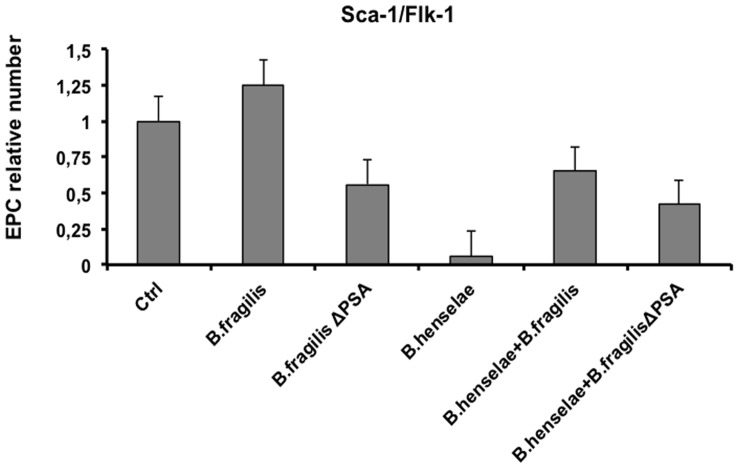
EPC number of infected mice. EPC relative number of infected mice *versus* control mice was measured by FACS analysis. Sca-I/Flk-I double positive cells were considered as murine EPCs. Bars indicate standard errors.

At 36 days after infection, before mice sacrifice, blood was collected in order to establish whether the EPC number was influenced by infection in all the studied groups. As shown in Figure 7, the mean number of EPCs, measured as Sca-I/Flk-I positive cells, dramatically decreased of about 90% in mice infected with *B. henselae* (0.06) compared to the uninfected group, whose EPC number was considered as 100% (1.0). Means were calculated by standard error method. This number was partially restored in mice coinfected with *B. fragilis* or *B. fragilis* ΔPSA and *B. henselae* (0.65 and 0.42 means respectively).

Thoroughly, these results indicate that *B. fragilis*, but not *B. fragilis* ΔPSA, is able to prevent the decrease of EPC induced by *B. henselae* infection.

## Discussion

The results of this study establish that: 1) *B. fragilis* is able to adhere and invade EPCs; 2) *B. fragilis* competes with *B. henselae* during *in vitro* coinfection assays; 3) the immunomodulatory properties of *B. fragilis* depend on PSA; 4) damages induced by *B. henselae* in liver and aorta of infected mice can be prevented by colonization with *B. fragilis* but not with its mutant *B. fragilis* ΔPSA; 5) EPC impairment prompted by *B. henselae* can be counteracted by *B. fragilis* colonization.

Our *in vitro* studies indicate that *B. fragilis* is able to adhere and invade EPCs and it competes with *B. henselae* during coinfection. Furthermore, we observed a marked activation of inflammatory response in *B. fragilis*-infected cells compared to *B. henselae*-infected, as determined by Real time PCR and ELISA of pro-inflammatory chemokines and cytokines. In the mouse model of coinfection, morphological and ultrastructural analyses showed that the damage induced by *B. henselae* in liver and aorta can be prevented in the coinfection with *B. fragilis* but not with its mutant *B. fragilis* ΔPSA. Specifically, the number of *B. henselae-*positive cells per field counted by immunohistochemistry analysis decreased of at least 50% in the liver, spleen and aorta sections of mice coinfected with both bacteria compared to mice infected only with *B. henselae*. This reduction was less evident in the tissue from mice coinfected with *B. fragilis* ΔPSA suggesting a possible role of the PSA also in this model. Finally, *B. fragilis* colonization was also able to restore the EPC number decrease observed in mice infected with *B. henselae*.

The findings of this complex study were achieved by performing a great bulk of experiments *in vitro* as well as *in vivo*. We have previously demonstrated that *B. henselae* internalizes EPCs [9], [29], also documenting the gene expression changes occurring during infection [9], [30]. A further study indicated that EPCs can be also a potential reservoirs of Kaposi's sarcoma-associated herpesvirus [31], thus extending our findings to other microorganisms. Noteworthy, this is the first study demonstrating that also *B. fragilis* can adhere to and invade EPCs as shown by confocal observations. Moreover, its internalization is not fully dependent on PSA since also the mutant ΔPSA strain exhibits such ability. This important finding has been achieved both *in vitro* on EPCs by confocal microscopy (Figure 1) and *in vivo* on infected mice tissues analyzed by ultrastructural analysis (Figure 6 and S6). To our knowledge, TEM examination has been used only in a few *in vitro* studies to explore cell internalization of *B. fragilis* but never for *B. fragilis* ΔPSA [32], [33]. A previous study showing the ability of *B. fragilis* PSA to stimulate an anti-inflammatory background in the intestine suggested that PSA may be critical for the establishment of a commensal intestinal flora [34]. Indeed, colonization experiments indicated that *B. fragilis* ΔPSA could not colonize the colonic epithelium as efficiently as the wild type strain [34]. Such different capability in tissue colonization has suggested that PSA may function as an adherence factor for *B. fragilis*, or reflect the immunomodulatory properties of PSA [18]. The protective role of *B. fragilis*, recently shown to be sufficient to correct some immune system deficiencies in the absence of bacterial colonization, and also to prevent intestinal inflammation in animal models of colitis [19], [20].

Our results corroborate the latter hypothesis. Indeed, on one side, both strains are able to adhere and invade cells, independently of the presence of PSA; on the other side, we confirmed the PSA immunomodulation ability through the analysis of inflammatory chemokine/cytokine during *in vitro* infection of EPCs.

In our previous study [9], we also showed the detrimental effects of *B. henselae* on EPC number as well as the deregulation of inflammatory gene networks after *in vitro* infection [30]. Here, we document *in vivo* the *Bartonella*-induced damage in the tissues of infected mice, also showing for the first time a dramatic decrease in EPC number compared to uninfected control mice. Interestingly, *B. fragilis* colonization was also able to restore the EPC number decrease occurring in mice infected with *B. henselae* (0.65 *vs* 0.06 means).

The absence of a marked inflammatory response evidenced by TEM in mice coinfected with *B. fragilis* and the presence of granulomas in the liver as well as of cells of the immune system in the adventitia of mice infected with only *B. henselae* are all signs of the protective effect of the PSA molecule of *Bacteroides*. This effect was also evidenced by a weaker inflammatory response in mice coinfected with *B. fragilis* ΔPSA. This finding suggests that even other molecules could be involved in the process of immunoregulation and exert a protective effect.

Moreover, a previous paper has shown that toxins of *B. fragilis* modify the actin cytoskeleton inducing disruption of intercellular junctions [35]. However, we have seen cellular junctions perfectly arranged between the liver cells (data not shown); this result indirectly demonstrates the inability of these bacteria to invade the liver cells, as observed in our TEM data where bacteria are seen only in endothelial cells or in the space of Disse.

In coinfected mice we also documented a decreased presence of granulomas in liver and minimal changes in aorta, thus demonstrating both the tissue damage reduction and a lower presence of *B. henselae* within the cells, with a consequent decrease in its cytopathic effect. In few granulomas we found a lower extension and an intracellular colocalization at the immunofluorescence with *B. fragilis*. According to colocalization experiments, we can assess that the expression of granulomas is due to a protective effect of *B. fragilis* that competes with *B. henselae* for adhesion sites, leading to a lower cytopathic effect, reduced necrosis and impaired release of pro-inflammatory molecules, resulting in a global protective effect. We can therefore conclude that the reduced amount of infected cells in coinfected mice leads to impaired cellular suffering, occurring in the few cells where both bacteria colocalize and where *B. henselae*-induced cytotoxic effect is exhibited. The lower cytotoxic effect is likely to be a direct consequence of the reduced expression of several inflammatory molecules and, in turn, an impaired inflammatory response (immunohistochemical data). Such process is usually related with the pathogenicity of a bacterial agent. The increase of the cell damage induced by *B. henselae* is shown in our model by an inflammatory infiltrate that is more conspicuous in the liver.

Taking together our data strongly indicate that *B. fragilis* protects from *B. henselae* induced damages, including vascular ones. This relevant finding may provide a basis for the development of novel therapies against such infections.

## Materials and Methods

### Bacterial Strains and Growth Conditions

The *B. henselae* strain ATCC49882 (LGC Promochem) was stored at −80°C in tryptone soya broth USP (TSB) (Oxoid) until use, and grown on Columbia agar supplemented with 5% defibrinated sheep blood (Oxoid) in a humidified atmosphere at 37°C and 5% CO_2_ for seven days. *B. fragilis* NCTC9343 and its mutant *B. fragilis* ΔPSA were kindly provided by Professor Dennis L. Kasper. The strains were stored at −80°C in brain heart infusion broth (Oxoid) until use, and grown anaerobically onto Schaedler agar with 5% sheep blood at 37°C for 48 h.

### EPC Isolation and Cultivation

Human EPCs were isolated from total peripheral blood mononuclear cells (PBMCs) as previously described in detail [9], [36], [37]. Written informed consent was obtained from donors according to the Declaration of Helsinki.

### *In vitro* EPC Infections

For *in vitro* infection experiments, *B. henselae*, *B. fragilis* and *B. fragilis* ΔPSA stock solutions were thawed, washed, suspended in antibiotic-free cell culture medium. Cultured EPCs were infected with *B. henselae* at multiplicity of infection (MOI) of 100 [9]. *B. fragilis* or *B. fragilis* ΔPSA were used at MOI of 100, 200, 400 to establish optimal concentrations *in vitro*. Bacteria were sedimented onto 3 days cultured EPCs by centrifugation at 1800×g for 5′ at room temperature (RT). Following 24–48–72 h of incubation the EPCs were infected with *B. fragilis* or *B. fragilis* ΔPSA and/or *B. henselae*. Coinfection with *B. henselae* was performed either at the same time or after 3 h of *B. fragilis* or *B. fragilis* ΔPSA infection. The cultures were stopped at the established time and EPCs were washed three times with phosphate buffer solution (PBS) to remove non-adherent bacteria. For selective removal of extracellular bacteria, 100 µg/ml gentamicin (Schering-Plough) was added. To determine CFU [9], the cells were washed 2 h with PBS after addition of gentamicin, lysed by the addition of ice-cold distilled water, and incubated for 5′ on ice. The MOI used for each experiment was confirmed by plating serial dilutions.

### Confocal Immunofluorescence Microscopy

Cultured cells were fixed with 3% paraformaldehyde for 20′ at RT and permeabilized with 0.1%Triton-X100. Cells were then incubated 60′ at RT with specific antibodies. Anti-*B. henselae* primary antibody (Novus Biologicals), diluted 1/100, was then revealed with secondary antibodies conjugated to Alexa Fluor 488 (1∶500; Invitrogen, Carlsbad, CA) for 30′ at RT. Anti-*B. fragilis* Lipopolysaccharides (LPS) primary antibody (Santa Cruz Biotechnology, INC.) was diluted 1/250 and revealed with secondary antibody conjugated with tetramethylrhodamine isothiocyanate, diluted 1/20 (TRITC, Dako). Microscopy was performed using Zeiss LSM 510 confocal microscope equipped with a plan-apochromat x40 (NA 1.4) oil immersion objective and cropped to higher magnification. Fluorescence emissions were acquired in multi-track mode using LP 505 filter. To minimize noise, each image was averaged three times.

### Measurement of Cytokines in Cell Culture Supernatants

Cell-free supernatants were collected from infected EPCs and assayed for levels of cytokines and chemokines by commercial enzyme-linked immunosorbent assay (ELISA) kits according to the manufacturer’s instructions (SABiosciences A Qiagen Company). The concentrations of cytokines (pg/ml) IL1A, IL 1B, L2, IL4, IL6, IL8, IL10, IL12, IL17A, IFNγ, TNFα and GM-CSF were measured using an ELISA reader (Tecan). Results are presented as mean error of the values ± standard mean. The Student’s t test was used for the comparison of means, and regression analysis was used to evaluate any correlation between different cytokine levels. A P value of <0.05 was considered significant.

### RNA Isolation and Quantitative Real-Time PCR Assays

Total RNA was isolated from *in vitro* cultured EPCs infected with *B. henselae*, *B. fragilis*, *B. fragilis* ΔPSA and uninfected, using standard TRIZOL (Invitrogen) protocol as previously described [37], [38]. Integrity and concentration were evaluated by gel electrophoresis and Nanodrop. The RNA quality of each sample was good, with 260/280 ratio in the range between 1.8 and 2.0. All purified RNAs were then converted into cDNA using the RT^2^ First Strand Kit (SABiosciences, Frederick, USA). Resulting cDNAs were used for the quantitative Real-Time PCR assays performed on a specific RT^2^ Profiler™ PCR Arrays (SABiosciences) [26].

In particular, samples were analyzed on the 96-well plate Human Inflammatory Cytokines & Receptors RT^2^ Profiler PCR Arrays (PAHS-011). Such arrays provide a mix between the large-scale ability of microarrays to analyze gene expression and the quantitative sensitivity of Real-Time PCR assay, with highly reproducible results.

A total of 89 genes (including 5 house-keeping genes) was analyzed on this PCR-array for each sample. Briefly, cDNAs were independently added to the RT^2^ SYBR Green qPCR Master Mix (SABiosciences, Frederick, USA) and then spotted on 6 Human Inflammatory Cytokines & Receptors PCR-arrays. All the experimental procedure was performed according to the manufacturer’s instructions for the 7900HT Real Time PCR system (Applied Biosystems). Melting curves were generated after the amplification of 40 cycles.

Relative gene expression was measured by using the 2^−ΔΔCt^ method. Values of threshold cycle (Ct) were converted to 2^−Ct^ in order to be proportional to the amount of transcripts in the samples. Ct values ≥35 cycles were considered undetectable. In order to compare the samples among them, 2^−ΔCt^ were calculated by normalizing expression data by the mean of housekeeping genes (as specified for PCR-Arrays). For comparing gene expression data derived from the different experimental conditions, we calculated 2^−ΔΔCt^ values, obtained by normalizing experimental data by reference data (uninfected EPCs) [26]. Data derived from such analysis were also checked by using an Excel sheet downloaded from the manufacturer’s website (http://www.sabiosciences.com/pcrarraydataanalysis.php). For each PCR reaction, the Excel sheet allows data normalization correcting all Ct values by the average Ct values of constantly expressed house-keeping genes present on the array. In our experiments we observed that among the standard housekeeping genes, Hypoxanthine-guanine phosphoribosyltransferase (*HPRT*) was highly variable among samples, thus it was not considered for further normalization of data. Differentially expressed genes were defined as those with at least 2-fold change variation.

### Ethics Statement

The mice were fed with laboratory food pellets and tap water *ad libitum*, and were bred and housed under specific pathogen free conditions at CEINGE-Advanced Biotechnologies, Naples, Italy. All animal experiments were carried out according to institutional guidelines. All efforts were made to minimize animal suffering and to reduce the number of mice used, in accordance with the European Communities Council Directive of November 24, 1986 (86/609/EEC). The protocol of the study has been reviewed and approved by Ethical Animal Care and Use Committee “Federico II” University of Naples, Prot. 2012/0085083.

### Murine Strains and Infection

Specific pathogens free (SPF) C57BL/6J, female mice 8 weeks of age, were purchased from Charles River Laboratory. *B. fragilis* or *B. fragilis* ΔPSA was given to animals in concentration 10^9^ CFU in 0.1 ml of PBS per mouse by oral administration. Mouse enteric colonization was initiated 7 days prior to *B. henselae* infection, and the bacterial suspension was given 4 times on alternate days. *B. henselae* was prepared as described above and thawed on ice prior to infection. Mice were infected with *B. henselae via* intraperitoneal route at dose of 10^9^ CFU/mouse suspended in TSB. Control mice were injected with the same medium in the absence of bacteria. Following infection, mice were observed daily for signs of illness. At indicated time after infection (36 days) blood was withdrawn by cardiac puncture before sacrifice and collected in heparinized tubes. At the times of euthanasia, liver, spleen and aorta were excised and fixed for molecular and microscopic analysis.

### Detection of *B. henselae* and *B. fragilis* DNA by PCR

To determine the presence of bacterial DNA in mouse tissue, we developed a nested-PCR assay to amplify a fragment of the *B. henselae gltA* gene (outer: 674 bp; inner: 353 bp) [28] and *B. fragilis frdA* gene (outer: 599 bp; inner: 354 bp). Slices of infected liver tissue (20 µm thick), from paraffin-embedded organs, were provided by the Comparative Pathology Facility of CEINGE-Advanced Biotechnologies. DNA was isolated by using the QIAamp tissue extraction kit (Qiagen, Hilden, Germany) according to the manufacturer’s protocol.

PCR was performed with AmpliTaq Gold polymerase (Applied Biosystems) in a volume of 50 µl in two sequential tubes. Following initial denaturation for 4′ at 95°C, amplification with outer primers was performed with 30 cycles of 1′ at 94°C, 1′ at 70°C, and 2′ at 72°C. Amplification with inner primers consisted of 30 cycles of 1′ at 94°C, 10′ at 30°C, and 1′ at 72°C. The final extension step was extended to 10′ in both rounds of PCR. Several negative controls were included to exclude cross-contamination [28]. The expected amplicons were visualized by electrophoresis in an agarose gel and by staining with ethidium bromide. The expected amplicons were detected by using two primer pairs: OuterBh R/L (674 bp), InnerBh R/L (353 bp); OuterBf R/L (599 bp), InnerBf R/L (353 bp) (Supporting information Table S1); due to the small number of bacterial cells in the analyzed sample, the Outer amplicons are not detectable by electrophoresis. The assay confirmed the presence of *B. henselae*, *B. fragilis* and *B. fragilis* ΔPSA in infected and coinfected tissue samples. PCR was repeated twice from two different DNA liver samples.

### Immunohistochemistry

All samples (liver, spleen and aorta) for immunohistochemistry were fixed in formaldehyde (4%), embedded in paraffin and sliced into 5 µm thick sections were cut; one slide was stained with haematoxylin-eosin to verify the presence of a sufficient amount of bacterial cells and the remaining were used for IHC analyses. After de-paraffinization, slides containing two serial sections were processed for heat-induced epitope retrieval. When cooled, slides were washed in PBS and then incubated for 60′ at 37°C with the primary antibody. Arterial segments, livers and spleens were stained with a mouse monoclonal antibody anti-*B. henselae* diluted 1/100 (Novus Biologicals). After a further wash in PBS, sections were incubated with the secondary antibody biotinylated for 15′ at 37°C. Epitopes recognized by the primary antibody were detected by an avidin-biotin-peroxidase method [39]. A count of positive cells per field (at 206× magnification) was made and 20 fields per each section were analyzed. The relative means and standard deviation (±SD) were calculated.

### Immunofluorescence

A number of staining procedures were available for localizing antigen-antibody reaction in tissue by fluorescence. Combination of either fluorescein- and rhodamine-conjugated antibodies in a single solution using the direct staining method will simultaneously detect two antigens in the same section. Liver tissues were collected, preserved and fixed as for immunofluorescence staining, were incubated with the two antisera in two different times (two-step method). Briefly, sections were deparaffinized and then applied normal serum. Samples were incubated with *B. henselae* primary antibody (Novus Biologicals), diluted 1/100, and washed in TRIS-HCl buffer. Samples were incubated with secondary antibody conjugated with tetramethylrhodamine isothiocyanate, diluted 1/20 (TRITC, Dako) and then washed. Sections were incubated with *B. fragilis* LPS primary antibody (Santa Cruz Biotechnology, INC.), diluted 1/250 washed in TRIS-HCL buffer, and incubated with secondary antibody conjugated with fluorescein isothiocyanate diluted 1/20 (FITC, Dako) and then washed. Antibodies incubation time was 1 h for each step. The observation was performed with a Confocal Microscope LSM 510 (Zeiss) by three independent observers.

### Transmission Electron Microscopy (TEM)

The aorta and liver tissues were cut into 1 mm^3^ blocks and fixed in 1.5% glutaraldehyde in 0,067 M cacodylate buffer at pH 7.4 [40]. The samples were left in fixative for 3 h at 4°C and then washed in 0,134 M cacodylate buffer, pH 7.4, at 4°C. Then they were post-fixed in 1% osmium tetroxide in 0,067 M cacodylate buffer, pH 7.4, at 4°C for 1 h and after washed in 0,134 M cacodylate buffer, pH 7.4, at 4°C. Fixed tissues were dehydrated in ascending series of ethyl alcohol and then embedded in epon. Semi-thin (1,5 µm) sections of TEM embedded liver and aorta samples were cut with a glass knife for light microscopic observations. Sections were stained with 1% toluidine blue solution prepared in 1% sodium tetraborate buffer. Ultra-thin (50–80 nm) section were cut and stained first with uranyl acetate 3% in 50% ethyl alcohol and then with 2,6% lead citrate. These sections, loaded on 200 mesh grids, were observed in a JEM-1011 (JEOL) transmission electron microscope at 100 kV.

### EPC Number in Infected Mice

A single blood aliquot was collected by cardiac puncture from the ventricle under anaesthesia, with Tribromoethanol 240 mg/Kg, using appropriate size needles and collected into heparin-coated tubes. A volume of 100 µl blood was incubated for 30′ in the dark with Fluorescein isothiocyanate (FITC)-conjugated monoclonal antibody against mouse (Ly-6A/E) Phosphatidylinositol-anchored protein (Sca-1) (BD Pharmingen) and Phycoerythrin (PE)-conjugated antibody against mouse Fetal liver kinase 1 (Flk-1) (BD Pharmingen). Isotype-identical IgG antibodies were used as controls (BD Pharmingen) [41]–[43]. After incubation, erythrocytes were lysed using FACS lysing solution (Becton-Dickinson), washed with PBS and analysed on a FACScan (Becton-Dickinson) using CellQuest software. Each analysis included approximately 10000 events, referred to the total number of white cells.

## Supporting Information

Figure S1 **Bacteroides EPC internalization at different MOIs.** Confocal images of human early EPCs infected with *B. fragilis* and *B. fragilis* ΔPSA at 100, 200 and 400 MOI. Cells were stained with anti-Bacteroides (red) specific antibody after 48 h from infection.
(TIF)

Figure S2 **Bacteroides and Bartonella EPC coinfection.** EPCs coinfected with *B. henselae,* and *B. fragilis* or *B. fragilis* ΔPSA as indicated. A MOI of 100 was used for all bacteria strains. Cells were stained with anti-Bacteroides (red) and with anti-Bartonella (green) specific antibodies after 24 h, 48 h and 72 h from infection.
(TIF)

Figure S3 **Differential expression of inflammatory genes in infected EPCs.** Percentage of differentially expressed inflammatory genes after EPC infection with *B. henselae, B. fragilis, B. fragilis* ΔPSA, *B. henselae* and *B. fragilis, B. henselae* and *B. fragilis* ΔPSA respectively, compared to uninfected EPCs.
(TIF)

Figure S4 **Levels of secreted inflammatory cytokines in infected EPCs measured by ELISA.** Bar graph showing the ELISA results from an array of 12 secreted cytokines involved in the inflammation response.
(TIF)

Figure S5 **Morphological analysis of murine infected spleen by hematoxylin-eosin and immunohistochemistry. A.** Representative microscope images of hematoxylin-eosin staining of spleen tissues from each group of mice uninfected, infected with *B. henselae*, *B. fragilis* and *B. fragilis* ΔPSA or coinfected, as detailed. **B.** Immunohistochemistry analysis of murine spleen samples with an antibody against *B. henselae* is shown (206× magnification). Bar graphs show the mean of the positive cell number per field (HPF) as indicated.
(TIF)

Figure S6 **Electron microscopy analysis of liver (panels A–E) and aorta (panels F–L) from infected mice. A.** *B. fragilis* (arrow). **A_I._** Detail of panel A. **B.** *B. fragilis* ΔPSA (arrow). **B_I._** Sinusoidal Kupffer cell with evident phagolysosome. **C.** *B. henselae* (arrow) in a granuloma with a characteristic neutrophil. **C_I_.** *B. henselae* in the endothelial layer. **D.** Bacterium in the sub-endothelial layer of liver coinfected with *B. henselae* and *B. fragilis* (arrow). **D_I_.** Detail of figure D. **D_II_.** Macrophage around centrilobular vein. **E.** Bacterium in the sub-endothelial layer of liver coinfected with *B. henselae* and *B. fragilis* (arrow). **E_I_.** Detail of image E. **F.** *B. fragilis* in the tunica intima (arrow); **F_I_.** *B. fragilis* in the tunica media (arrow). **G.** *B. fragilis* ΔPSA in the tunica intima (arrow). **G_I_.** *B. fragilis* ΔPSA in the tunica media (arrow). **H.** *B. henselae* in the tunica intima (arrow). **H_I_.** *B. henselae* in the tunica media (arrow). **H_II_.** Swollen endothelium of aorta infected with *B. henselae*. **I.** Bacterium in the tunica intima of aorta coinfected with *B. henselae* and *B. fragilis* (arrow). **I_I_.** Bacteria in the tunica media of aorta coinfected with *B. henselae* and *B. fragilis* (arrow). **J.** Bacteria in the tunica intima of aorta coinfected with *B. henselae* and *B. fragilis* ΔPSA (arrow). **J_I_.** Bacteria in the tunica media of aorta coinfected with *B. henselae* and *B. fragilis* ΔPSA (arrow).
(TIF)

Table S1 **Oligonucleotides used in this study.**
(DOC)
