# Supplementary figures and images for: Evidence of Bacteroides fragilis Protection from Bartonella henselae-Induced Damage
Source: PLoS One. 2012 Nov 15;7(11):e49653. doi: 10.1371/journal.pone.0049653 (PMC3499472; doi:10.1371/journal.pone.0049653)

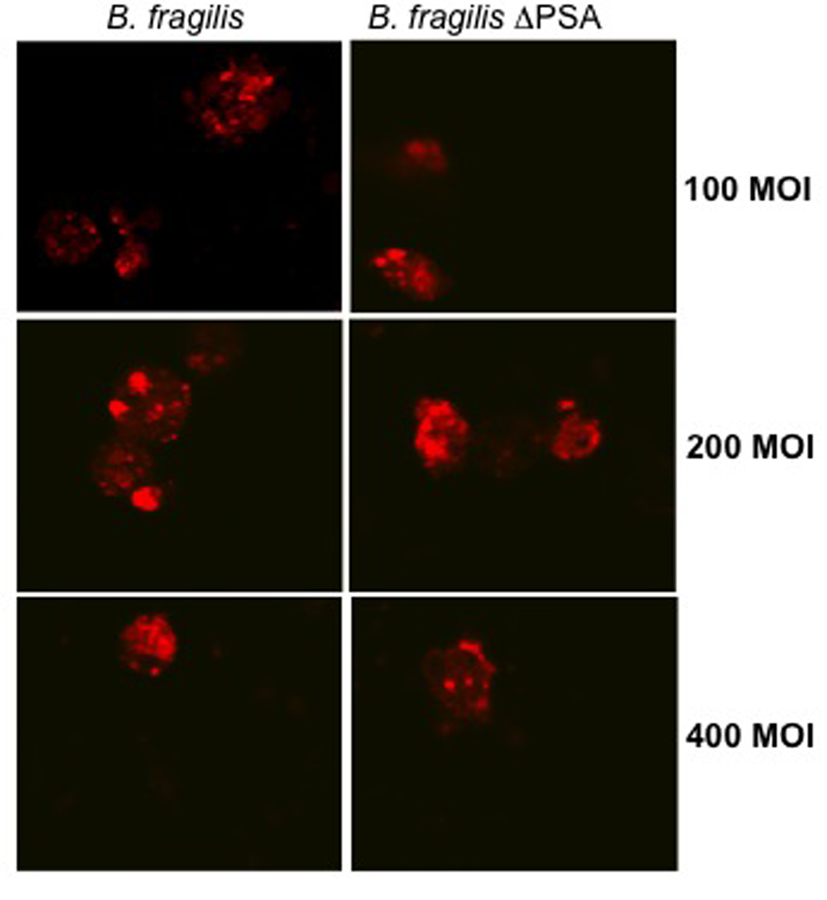

Supplement: Figure S1 — Bacteroides EPC internalization at different MOIs. Confocal images of human early EPCs infected with B. fragilis and B. fragilis ΔPSA at 100, 200 and 400 MOI. Cells were stained with anti-Bacteroides (red) specific antibody after 48 h from infection. (TIF) [file pone.0049653.s001.tif]

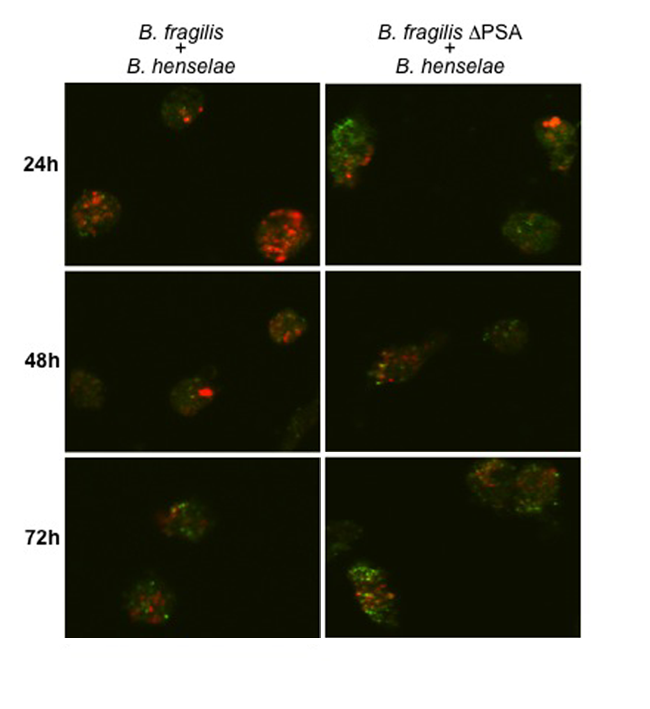

Supplement: Figure S2 — Bacteroides and Bartonella EPC coinfection. EPCs coinfected with B. henselae, and B. fragilis or B. fragilis ΔPSA as indicated. A MOI of 100 was used for all bacteria strains. Cells were stained with anti-Bacteroides (red) and with anti-Bartonella (green) specific antibodies after 24 h, 48 h and 72 h from infection. (TIF) [file pone.0049653.s002.tif]

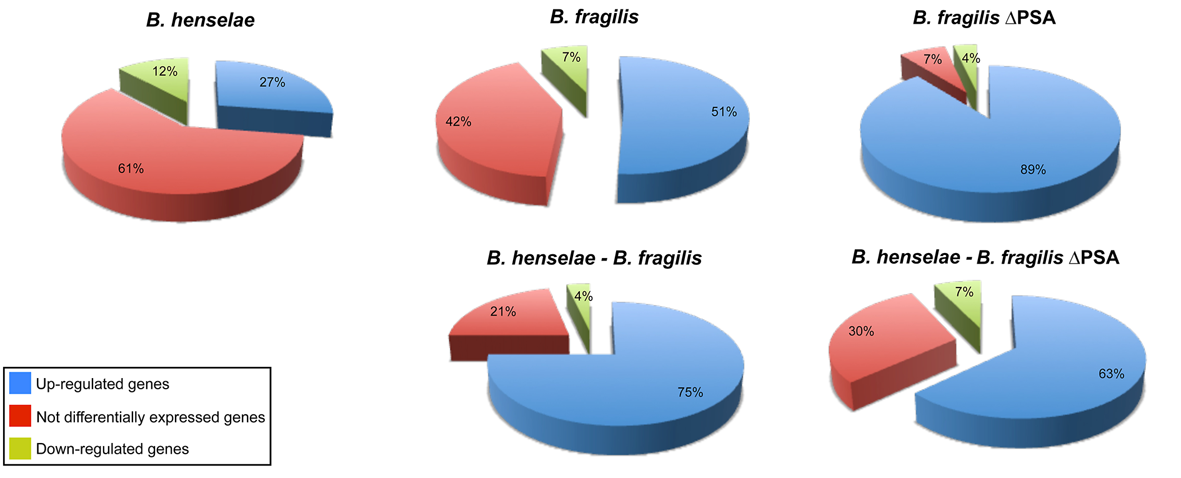

Supplement: Figure S3 — Differential expression of inflammatory genes in infected EPCs. Percentage of differentially expressed inflammatory genes after EPC infection with B. henselae, B. fragilis, B. fragilis ΔPSA, B. henselae and B. fragilis, B. henselae and B. fragilis ΔPSA respectively, compared to uninfected EPCs. (TIF) [file pone.0049653.s003.tif]

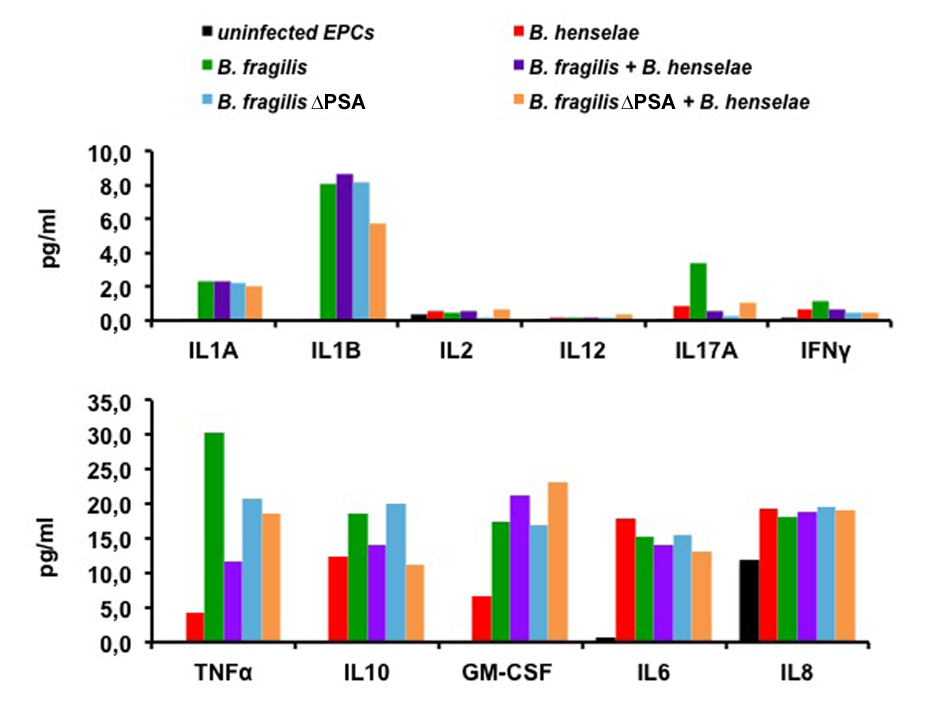

Supplement: Figure S4 — Levels of secreted inflammatory cytokines in infected EPCs measured by ELISA. Bar graph showing the ELISA results from an array of 12 secreted cytokines involved in the inflammation response. (TIF) [file pone.0049653.s004.tif]

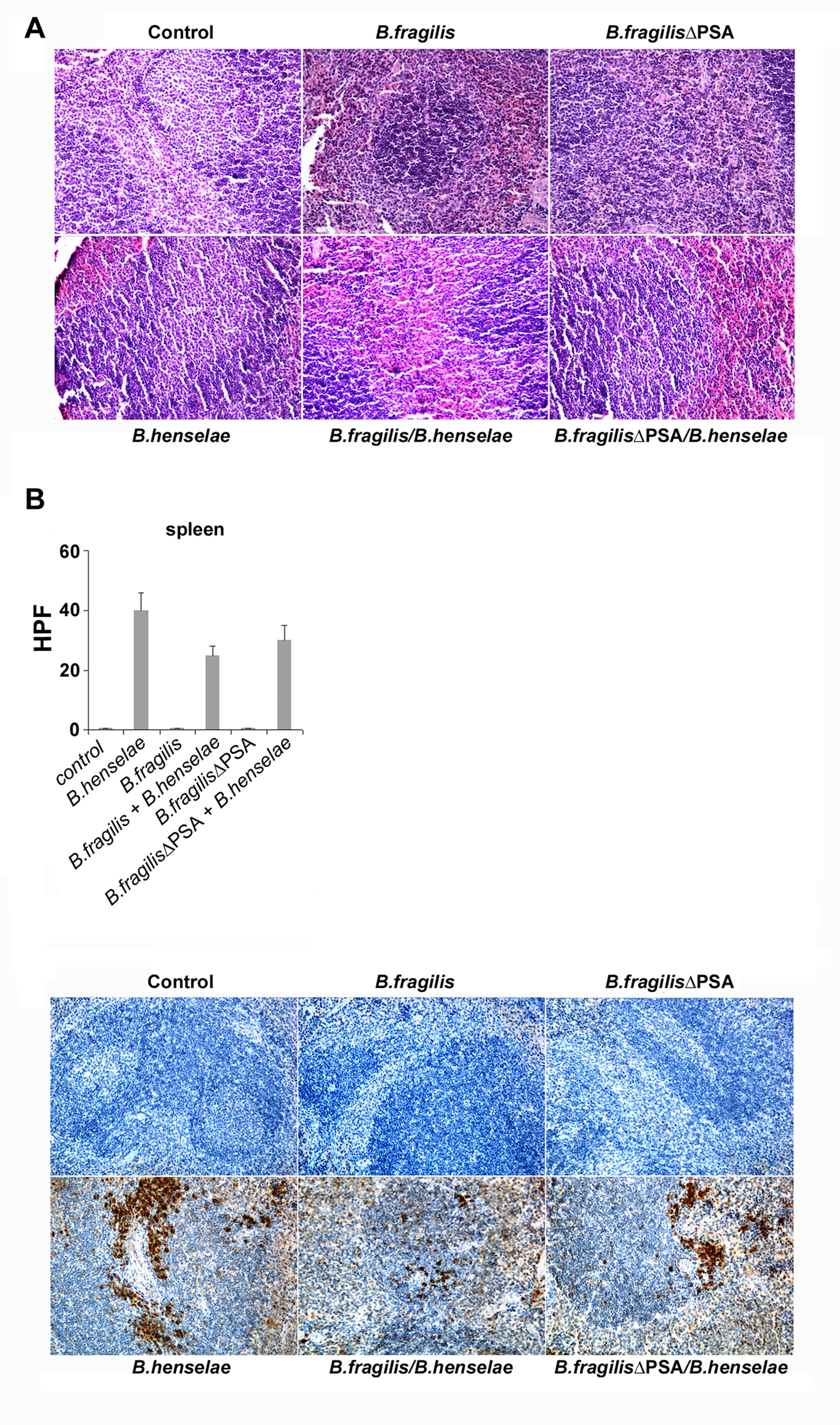

Supplement: Figure S5 — Morphological analysis of murine infected spleen by hematoxylin-eosin and immunohistochemistry. A. Representative microscope images of hematoxylin-eosin staining of spleen tissues from each group of mice uninfected, infected with B. henselae, B. fragilis and B. fragilis ΔPSA or coinfected, as detailed. B. Immunohistochemistry analysis of murine spleen samples with an antibody against B. henselae is shown (206× magnification). Bar graphs show the mean of the positive cell number per field (HPF) as indicated. (TIF) [file pone.0049653.s005.tif]

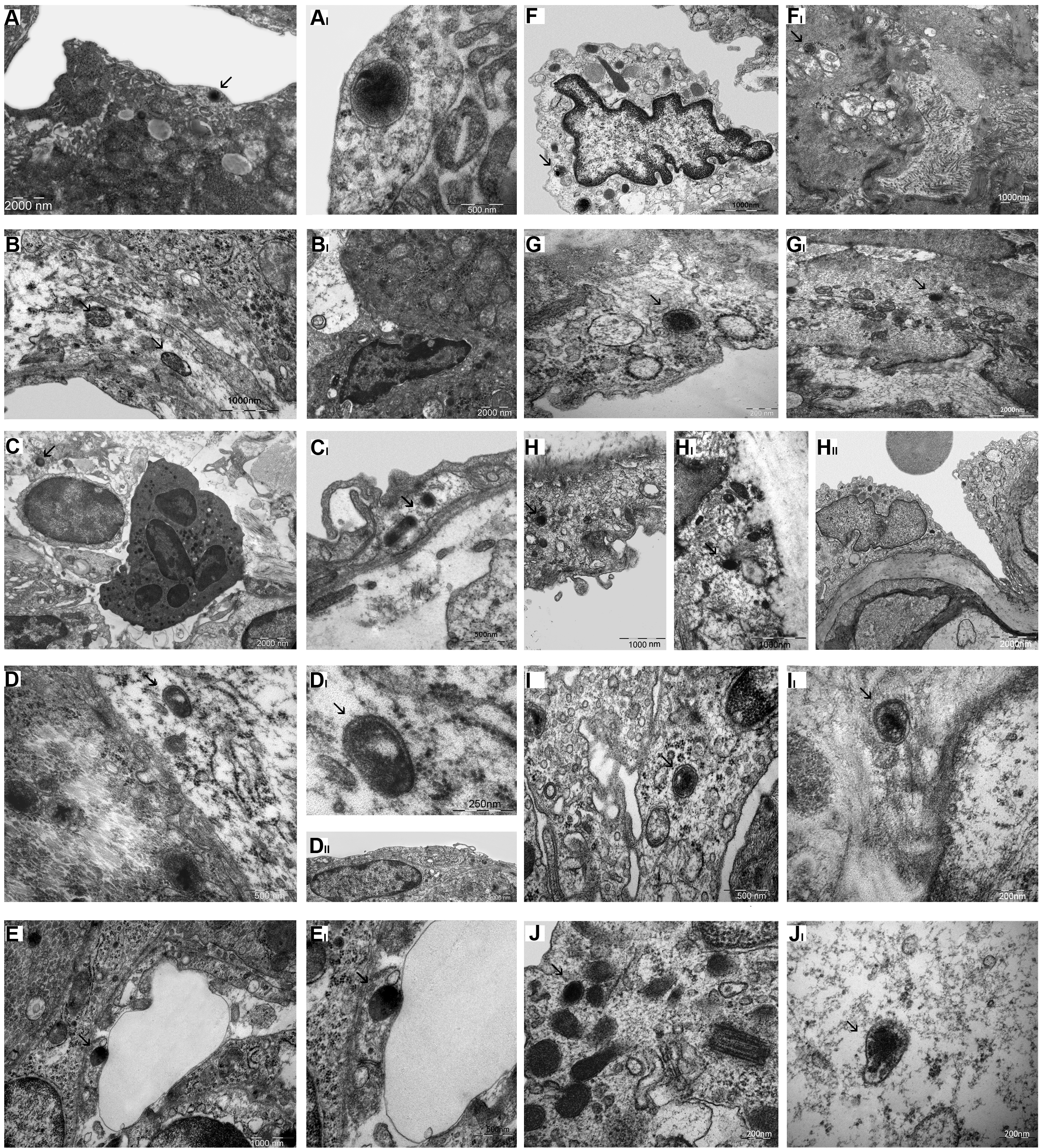

Supplement: Figure S6 — Electron microscopy analysis of liver (panels A–E) and aorta (panels F–L) from infected mice. A. B. fragilis (arrow). AI. Detail of panel A. B. B. fragilis ΔPSA (arrow). BI. Sinusoidal Kupffer cell with evident phagolysosome. C. B. henselae (arrow) in a granuloma with a characteristic neutrophil. CI. B. henselae in the endothelial layer. D. Bacterium in the sub-endothelial layer of liver coinfected with B. henselae and B. fragilis (arrow). DI. Detail of figure D. DII. Macrophage around centrilobular vein. E. Bacterium in the sub-endothelial layer of liver coinfected with B. henselae and B. fragilis (arrow). EI. Detail of image E. F. B. fragilis in the tunica intima (arrow); FI. B. fragilis in the tunica media (arrow). G. B. fragilis ΔPSA in the tunica intima (arrow). GI. B. fragilis ΔPSA in the tunica media (arrow). H. B. henselae in the tunica intima (arrow). HI. B. henselae in the tunica media (arrow). HII. Swollen endothelium of aorta infected with B. henselae. I. Bacterium in the tunica intima of aorta coinfected with B. henselae and B. fragilis (arrow). II. Bacteria in the tunica media of aorta coinfected with B. henselae and B. fragilis (arrow). J. Bacteria in the tunica intima of aorta coinfected with B. henselae and B. fragilis ΔPSA (arrow). JI. Bacteria in the tunica media of aorta coinfected with B. henselae and B. fragilis ΔPSA (arrow). (TIF) [file pone.0049653.s006.tif]
