# Supplementary material for: Evidence of Bacteroides fragilis Protection from Bartonella henselae-Induced Damage
Source: PLoS One. 2012 Nov 15;7(11):e49653. doi: 10.1371/journal.pone.0049653 (PMC3499472; doi:10.1371/journal.pone.0049653)
Supplement: Table S1 — Oligonucleotides used in this study. (DOC) [file pone.0049653.s007.doc]

**SUPPLEMENTARY INFORMATION**

**TABLE S1.** Oligonucleotides used in this study

| **Name** | **Sequence** |
| --- | --- |
| Outer Bh R | 5’GGT CCC AAC TCT TGC CGC TAT G 3’ ^[38]^ |
| Outer Bh L | 5’CAG CCG ACA CTG CGT GCT AAT G 3’. ^[38]^ |
| Outer Bf R | 5’AAG CCA ACG TTT ACC GTC TGG C 3’ |
| Outer Bf L | 5’TAG TGC CTG CCT GCA ATG CTT TGG 3’ |
| Inner Bh R | 5’ATG CCT AAA AAT GTT ACA AGA 3’^[38]^ |
| Inner Bh L | 5’CGT GCT AAT GCA AAA AGA AC 3’^[38]^ |
| Inner Bf R | 5’ACC GTC TGG CTG AGG TAT CAA ACG 3’ |
| Inner Bf L | 5’TGC GTT TCC GTA ACC ACC TGT ACC 3’ |
